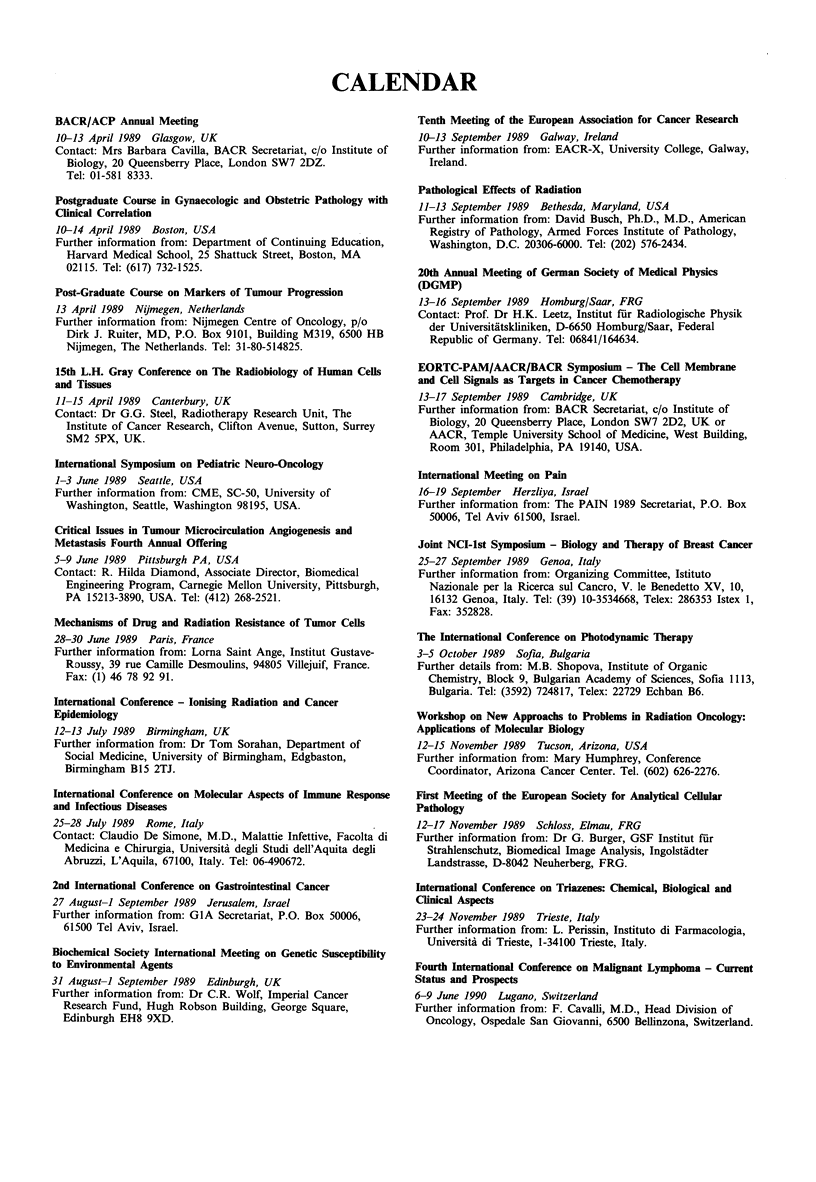# Calendar

**Published:** 1989-04

**Authors:** 


### CALENDAR

**BACR/ACP Annual Meeting**

*10–13 April 1989 Glasgow, UK*

Contact: Mrs Barbara Cavilla, BACR Secretariat, c/o Institute of Biology, 20 Queensberry Place, London SW7 2DZ. Tel: 01-581 8333.

**Postgraduate Course in Gynaecologic and Obstetric Pathology with Clinical Correlation**

*10–14 April 1989 Boston, USA*

Further information from: Department of Continuing Education, Harvard Medical School, 25 Shattuck Street, Boston, MA 02115. Tel: (617) 732-1525.

**Post-Graduate Course on Markers of Tumour Progression**

*13 April 1989 Nijmegen, Netherlands*

Further information from: Nijmegen Centre of Oncology, p/o Dirk J. Ruiter, MD, P.O. Box 9101, Building M319, 6500 HB Nijmegen, The Netherlands. Tel: 31-80-514825.

**15th L.H. Gray Conference on The Radiobiology of Human Cells and Tissues**

*11–15 April 1989 Canterbury, UK*

Contact: Dr G.G. Steel, Radiotherapy Research Unit, The Institute of Cancer Research, Clifton Avenue, Sutton, Surrey SM2 5PX, UK.

**International Symposium on Pediatric Neuro-Oncology**

*1–3 June 1989 Seattle, USA*

Further information from: CME, SC-50, University of Washington, Seattle, Washington 98195, USA.

**Critical Issues in Tumour Microcirculation Angiogenesis and Metastasis Fourth Annual Offering**

*5–9 June 1989 Pittsburgh PA, USA*

Contact: R. Hilda Diamond, Associate Director, Biomedical Engineering Program, Carnegie Mellon University, Pittsburgh, PA 15213-3890, USA. Tel: (412) 268-2521.

**Mechanisms of Drug and Radiation Resistance of Tumor Cells**

*28–30 June 1989 Paris, France*

Further information from: Lorna Saint Ange, Institut Gustave-Roussy, 39 rue Camille Desmoulins, 94805 Villejuif, France. Fax: (1) 46 78 92 91.

**International Conference – Ionising Radiation and Cancer Epidemiology**

*12–13 July 1989 Birmingham, UK*

Further information from: Dr Tom Sorahan, Department of Social Medicine, University of Birmingham, Edgbaston, Birmingham B15 2TJ.

**International Conference on Molecular Aspects of Immune Response and Infectious Diseases**

*25–28 July 1989 Rome, Italy*

Contact: Claudio De Simone, M.D., Malattie Infettive, Facolta di Medicina e Chirurgia, Università degli Studi dell'Aquita degli Abruzzi, L'Aquila, 67100, Italy. Tel: 06-490672.

**2nd International Conference on Gastrointestinal Cancer**

*27 August–1 September 1989 Jerusalem, Israel*

Further information from: G1A Secretariat, P.O. Box 50006, 61500 Tel Aviv, Israel.

**Biochemical Society International Meeting on Genetic Susceptibility to Environmental Agents**

*31 August–1 September 1989 Edinburgh, UK*

Further information from: Dr C.R. Wolf, Imperial Cancer Research Fund, Hugh Robson Building, George Square, Edinburgh EH8 9XD.

**Tenth Meeting of the European Association for Cancer Research**

*10–13 September 1989 Galway, Ireland*

Further information from: EACR-X, University College, Galway, Ireland.

**Pathological Effects of Radiation**

*11–13 September 1989 Bethesda, Maryland, USA*

Further information from: David Busch, Ph.D., M.D., American Registry of Pathology, Armed Forces Institute of Pathology, Washington, D.C. 20306-6000. Tel: (202) 576-2434.

**20th Annual Meeting of German Society of Medical Physics (DGMP)**

*13–16 September 1989 Homburg/Saar, FRG*

Contact: Prof. Dr H.K. Leetz, Institut für Radiologische Physik der Universitätskliniken, D-6650 Homburg/Saar, Federal Republic of Germany. Tel: 06841/164634.

**EORTC-PAM/AACR/BACR Symposium – The Cell Membrane and Cell Signals as Targets in Cancer Chemotherapy**

*13–17 September 1989 Cambridge, UK*

Further information from: BACR Secretariat, c/o Institute of Biology, 20 Queensberry Place, London SW7 2D2, UK or AACR, Temple University School of Medicine, West Building, Room 301, Philadelphia, PA 19140, USA.

**International Meeting on Pain**

*16–19 September Herzliya, Israel*

Further information from: The PAIN 1989 Secretariat, P.O. Box 50006, Tel Aviv 61500, Israel.

**Joint NCI-1st Symposium – Biology and Therapy of Breast Cancer**

*25–27 September 1989 Genoa, Italy*

Further information from: Organizing Committee, Istituto Nazionale per la Ricerca sul Cancro, V. le Benedetto XV, 10, 16132 Genoa, Italy. Tel: (39) 10-3534668, Telex: 286353 Istex 1, Fax: 352828.

**The International Conference on Photodynamic Therapy**

*3–5 October 1989 Sofia, Bulgaria*

Further details from: M.B. Shopova, Institute of Organic Chemistry, Block 9, Bulgarian Academy of Sciences, Sofia 1113, Bulgaria. Tel: (3592) 724817, Telex: 22729 Echban B6.

**Workshop on New Approachs to Problems in Radiation Oncology: Applications of Molecular Biology**

*12–15 November 1989 Tucson, Arizona, USA*

Further information from: Mary Humphrey, Conference Coordinator, Arizona Cancer Center. Tel. (602) 626-2276.

**First Meeting of the European Society for Analytical Cellular Pathology**

*12–17 November 1989 Schloss, Elmau, FRG*

Further information from: Dr G. Burger, GSF Institut für Strahlenschutz, Biomedical Image Analysis, Ingolstädter Landstrasse, D-8042 Neuherberg, FRG.

**International Conference on Triazenes: Chemical, Biological and Clinical Aspects**

*23–24 November 1989 Trieste, Italy*

Further information from: L. Perissin, Instituto di Farmacologia, Università di Trieste, 1-34100 Trieste, Italy.

**Fourth International Conference on Malignant Lymphoma – Current Status and Prospects**

*6–9 June 1990 Lugano, Switzerland*

Further information from: F. Cavalli, M.D., Head Division of Oncology, Ospedale San Giovanni, 6500 Bellinzona, Switzerland.